# Using the high-temperature phase transition of iron sulfide minerals as an indicator of fault slip temperature

**DOI:** 10.1038/s41598-019-44319-8

**Published:** 2019-05-28

**Authors:** Yan-Hong Chen, Yen-Hua Chen, Wen-Dung Hsu, Yin-Chia Chang, Hwo-Shuenn Sheu, Jey-Jau Lee, Shih-Kang Lin

**Affiliations:** 1 Department of Earth Sciences, National Cheng Kung University, 701 Tainan, Taiwan; 2 Department of Materials Science and Engineering, National Cheng Kung University, 701 Tainan, Taiwan; 3 National Synchrotron Radiation Research Center, 300 Hsinchu, Taiwan

**Keywords:** Geophysics, Mineralogy

## Abstract

The transformation of pyrite into pyrrhotite above 500 °C was observed in the Chelungpu fault zone, which formed as a result of the 1999 Chi-Chi earthquake in Taiwan. Similarly, pyrite transformation to pyrrhotite at approximately 640 °C was observed during the Tohoku earthquake in Japan. In this study, we investigated the high-temperature phase-transition of iron sulfide minerals (greigite) under anaerobic conditions. We simulated mineral phase transformations during fault movement with the aim of determining the temperature of fault slip. The techniques used in this study included thermogravimetry and differential thermal analysis (TG/DTA) and *in situ* X-ray diffraction (XRD). We found diversification between 520 °C and 630 °C in the TG/DTA curves that signifies the transformation of pyrite into pyrrhotite. Furthermore, the *in situ* XRD results confirmed the sequence in which greigite underwent phase transitions to gradually transform into pyrite and pyrrhotite at approximately 320 °C. Greigite completely changed into pyrite and pyrrhotite at 450 °C. Finally, pyrite was completely transformed into pyrrhotite at 580 °C. Our results reveal the temperature and sequence in which the phase transitions of greigite occur, and indicate that this may be used to constrain the temperature of fault-slip. This conclusion is supported by field observations made following the Tohoku and Chi-Chi earthquakes.

## Introduction

Iron sulfide minerals, which include marcasite (FeS_2_), pyrite (FeS_2_), greigite (Fe_3_S_4_), and pyrrhotite (Fe_7_S_8_), are common in the Earth’s crust. Iron sulfide minerals have many applications; for example, greigite, a ferrimagnetic mineral, can be used as a paleomagnetic indicator^1,2^ and for treating hyperthermia associated with cancer^3^, and pyrrhotite, which can be used as a geological thermometer^4,5^. Pyrite has photovoltaic and semiconducting properties owing to its favorable energy gap^6,7^. Different iron sulfide minerals can be formed during sediment deposition as a consequence of the prevailing redox conditions^1,2,8–11^. The surfaces of marine and lake sediments are oxic environments, however as the depth from the surface of the sediment increases, the environment becomes increasingly anaerobic. With increasing depth, the progression of deep-sea sedimentary environmental conditions is: nitrogenous, manganous, ferruginous, sulfidic, and methanic^12,13^. Iron sulfide minerals form in the sulfate-methane transition zone (SMTZ), which is the habitat for anaerobic methanotrophic archaea (ANME) and sulfate reducing bacteria (SRB). First, the ANME decompose methane to produce carbon dioxide, hydrogen, and acetate, and then the SRB reduce sulfate using hydrogen and acetate to produce hydrogen sulfide and bicarbonate. The hydrogen sulfide produced in this process subsequently reacts with ferrous ions to form iron sulfide minerals^12–14^. Additionally, iron sulfide minerals can be formed by precipitation from hydrothermal fluids, forming submarine black smoker chimneys^15,16^. Magnetotactic bacteria, which are similar to greigite-producing bacteria, can also produce greigite under anaerobic conditions^14^. Therefore, the presence of sulfide minerals is an indicator of an anaerobic environment.

Accurate measurement of fault-slip temperature is very difficult. The possible methods to measure fault-slip temperature include: use of the phase assemblages of clay minerals^17,18^, the thermal maturity of carbonaceous material^19,20^, the characterization of magnetic minerals^21,22^, high-speed rotary shear experiments^23,24^, among others. Greigite and pyrite are very stable under pressures of up to 3.4 GPa and 40 GPa^25,26^, respectively, which correspond to depths of ~125 km and ~1450 km beneath the earth’s surface, respectively. The epicenters of high magnitude earthquakes that have occurred in the last 30 years (e.g., Tohoku earthquake in Japan, Wenchuan earthquake in China, Chi-Chi earthquake in Taiwan, and Hanshin earthquake in Japan) were all located at depths less than 30 km, and are classified as shallow focus earthquakes. Offshore drilling research found that pyrite was converted into pyrrhotite as a result of high-temperature friction in the fault-slip zone in the offshore sediments of northeastern Japan (Tohoku earthquake)^27^. A study of the 1999 Chi-Chi earthquake in Taiwan also observed that high temperatures generated by the fault slip caused pyrite to transform into pyrrhotite^28^. Therefore, the high-temperature phase transition of iron sulfide minerals such as greigite (Fe_3_S_4_) may be another method for measuring fault slip temperature.

We therefore investigated the high-temperature phase transition of pyrite in nature, caused by fault slip, by designing a series of experiments in which greigite undergoes a high-temperature phase transition in an anaerobic environment. Our motivation for using greigite (rather than pyrite) as the starting material is that greigite is considered to be an intermediary in the conversion of mackinawite (FeS) to pyrite (FeS_2_)^29–32^. Although greigite is a metastable iron sulfide mineral, it has been found in a deep core from Qinghai Lake in China, where it was preserved in Pliocene-age sediments^2^. A study of sedimentary rocks from southwestern Taiwan also found a stratum containing magnetic minerals such as greigite and pyrrhotite^33^. Greigite is an interesting iron sulfide mineral, very few studies that discuss the greigite-pyrite-pyrrhotite transition under high-temperature conditions have been published. Therefore, our intent is to show that the sequence in which the phase transitions occur corresponds to our prediction, namely that greigite first transforms to pyrite, which is then converted to pyrrhotite. The second transformation in this sequence resembles the phase transition of pyrite as it occurs in nature when it is caused by friction due to fault slip.

## Methods

In this study, greigite was first synthesized via a hydrothermal method^34^. The phase transition temperature of greigite was then obtained by carrying out thermogravimetric and differential thermal analyses (TG-DTA). Moreover, the phase diagram of iron and sulfur was calculated by using Fact Sage software. The high-temperature phase transformation and the transformation pathway of greigite under anaerobic conditions was observed using synchrotron radiation *in situ* X-ray diffraction (XRD) measurements. The related details are as follows.

### Synthesis

Synthetic greigite was fabricated by hydrothermal synthesis according to the following procedure. The solution was deployed, filtered, and then stored in an anaerobic glove box. Iron (II) sulfate heptahydrate (FeSO_4_•7H_2_O) and thioacetamide (C_2_H_5_NS) were formulated into 0.2 and 0.3 M solutions, respectively, such that the volume of each was 50 mL. The solutions were then mixed and loaded into an autoclave. The anaerobic conditions in the autoclave were maintained by injecting nitrogen gas. The mixture was heated to 115 °C and held at this temperature for 2 h to fabricate greigite. After cooling, the solution was filtered with deionized water and an acetic acid filter and then dried in a freeze dryer for more than 12 h to obtain greigite powders.

### Analysis

The fabricated greigite was confirmed to consist of the pure phase by XRD (Bruker D8 Advance with a wavelength of Cu Kα, a scanning rate of 1°/min, and a scanning step of 0.02°; the working voltage was 30 kV and the current was 20 mA). Next, we observed the weight change and exothermic heat release reaction of the sample at high temperatures by TG-DTA (Netzsch STA 449 F3 at a heating rate of 10 °C/min, from 25 to 900 °C). We used 5N ultra-high-purity nitrogen and an oxygen trapping system to reduce the oxygen content in the chamber. Finally, the sequence of greigite phase changes was examined by *in situ* XRD with synchrotron radiation. The *in situ* XRD diffraction patterns were recorded in the BL01C2 and BL17A1 beamlines of the National Synchrotron Radiation Research Center (NSRRC) in Taiwan. The energy of the storage ring was 1.5 GeV and the current was 360 mA. The wavelength of the beamline was 1.03 Å and 1.32 Å for BL01C2 and BL17A1, respectively. The exposure time was 72/192s. Both beamlines used a MAR345 detector, which is a two-dimensional area detector. The original 2D data were integrated into a one-dimensional diffraction pattern by GSASII, and the wavelength was converted to Cu Kα by Winplot software for a clear comparison.

## Results

### XRD

**Figure 1:**
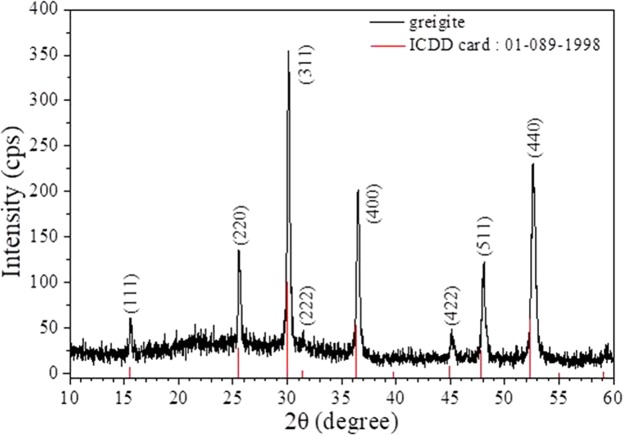
XRD pattern of synthetic greigite.

The diffraction peaks of the synthesized samples were compared to the values on the International Centre for Diffraction Data (ICDD) card 01-089-1998 (Fig. 1); however, the diffraction pattern was shifted to a slightly higher angle relative to that on the card. Greigite crystallizes in the cubic crystal system and the measured lattice parameter of the crystallographic axes is 9.845 Å (9.876 Å on the ICDD card). The observed differences in the pattern may have been due to defects resulting from the mismatch in the Fe^2+^/Fe^3+^ ratio in the greigite sample.

### TG-DTA

**Figure 2:**
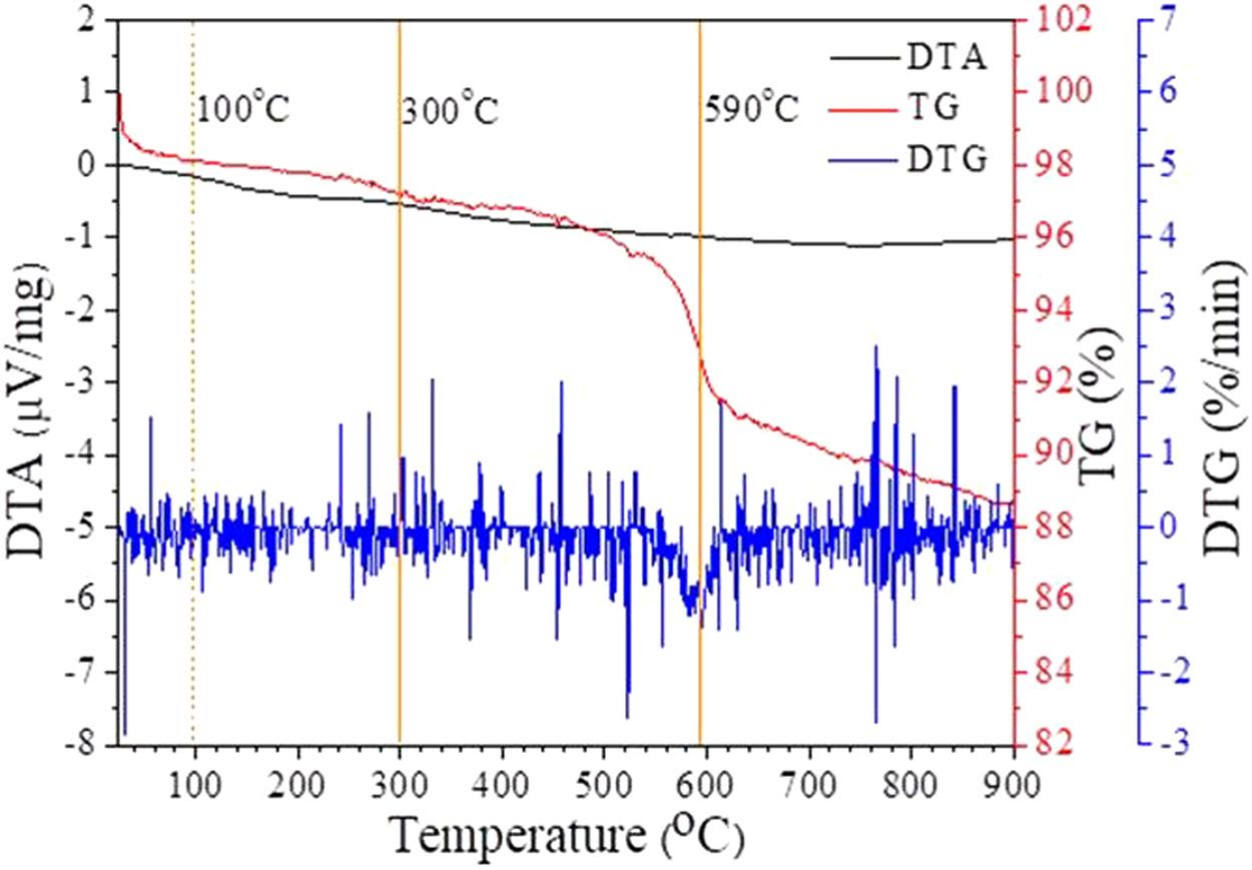
The TG/DTA curves of synthetic greigite.

**Figure 3:**
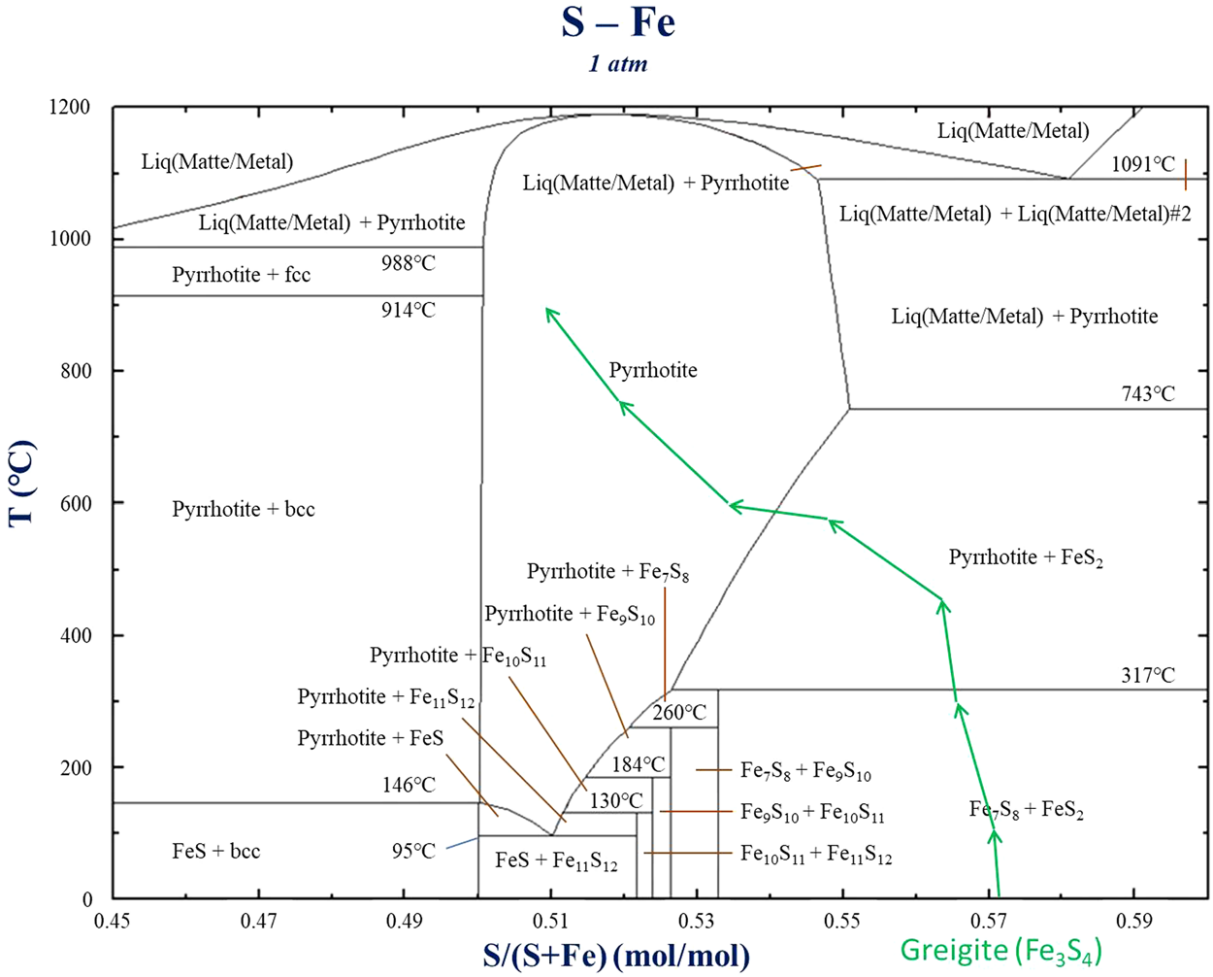
Phase diagram of iron sulfide mineral simulated using the Fact Sage software.

Figure 2 shows the TG-DTA results for greigite (Fe_3_S_4_). The DTA curve does not change significantly with temperature. The change in the TG curve below 100 °C is the result of desorption of water from the sample. The TG curve shows a small decrease at 300 °C and a large change at approximately 590 °C, indicating weight loss during heating. Assuming that the weight loss was the loss of sulfur from the iron sulfide mineral, the variation in the ratio between iron and sulfur can be calculated (as marked in Fig. 3 with green arrows).

### Phase diagram

Figure 3 presents the phase diagram of iron and sulfur calculated by Fact Sage software (the software algorithm^35,36^ is based on the well-known Gibbs-Helmholtz relation: G = H − TS, where H is enthalpy, S is entropy, and G is free energy calculated from this formula), with an ordinate temperature and abscissa of the molar ratio of sulfur in the iron sulfide mineral (sulfur accounts for 57.1% of greigite). Greigite can be replaced with a stable mineral in the phase diagram because it is a metastable mineral. The iron/sulfur ratio as a function of temperature was calculated according to the weight change observed in the TG-DTA experiment, which is marked on the figure with green arrows. The calculation result showed that greigite undergoes conversion to pyrite at 317 °C and is converted to pyrrhotite between 600 and 700 °C. The most stable mineral phase would exist at the lowest free energy based on this software-simulation concept. Therefore, the phase transformation occurred in the sequence of greigite → greigite + pyrrhotite + pyrite → pyrrhotite + pyrite → pyrrhotite, which is consistent with the TG-DTA results.

### *In situ* XRD

**Figure 4:**
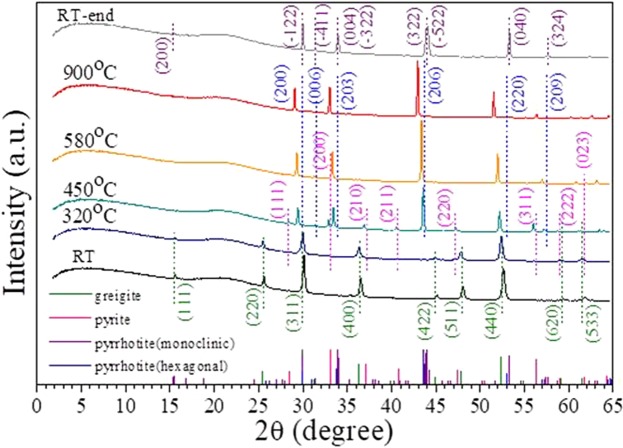
*In situ* XRD patterns of greigite and its phase transformation at various temperatures.

Figure 4 presents the XRD pattern of greigite and its phase transition at different temperatures. The pure phase of greigite was stable from room temperature to 300 °C. According to the figure, pyrite and pyrrhotite (hexagonal structure) began to appear at 320 °C. The (311), (400), and (511) diffraction peaks of greigite disappeared at approximately 450 °C, and the (200) and (023) diffraction peaks of pyrite disappeared at approximately 580 °C. Only the pyrrhotite phase remained when the temperature reached 900 °C. After returning to room temperature, the final product of the heating process, pyrrhotite (monoclinic structure), was present^37^. This result suggests that the high-temperature phase transition is irreversible, and that pyrrhotite can exist as a stable phase under anaerobic conditions.

### Comparison of natural and experimental behavior

**Figure 5:**
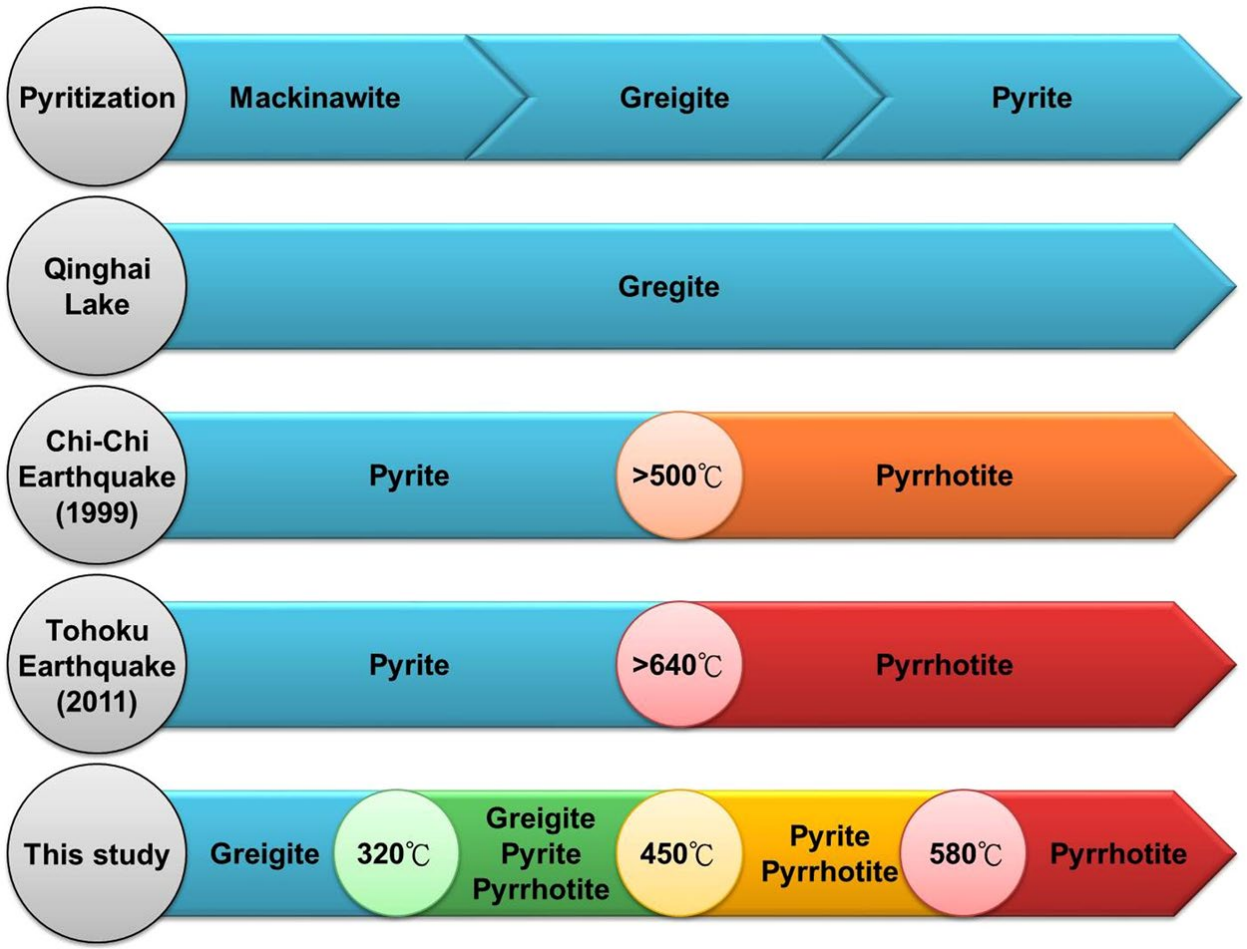
Diagram comparing the phase transition sequence and temperature of greigite, including pyritization, the preservation of greigite in Qinghai Lake, phase-change sequence during the Chi-Chi and Tohoku earthquakes, and the phase transformation of greigite determined in this study.

Figure 5 illustrates the formation of iron sulfide and the sequence of high-temperature phase transitions of greigite. Mackinawite is well known to be the initial precipitate of iron sulfide minerals during the reaction of Fe^2+^ with S^2−^ solutions. In a slightly oxidizing or hydrogen sulfide rich environment, mackinawite changes into greigite and then into pyrite (i.e., pyritization). There are a lot of locations where greigite was found, for example in Qinghai Lake (China)^2^, in mudstones (Taiwan)^19^, among others. The deposition rate in these areas^2,19^ was extremely rapid, thus the proportion of organic carbon and hydrogen sulfate in the sediments was diluted; this resulted in stagnant pyritization, causing the greigite to be preserved. A study of the 1999 Chi-Chi earthquake in Taiwan indicated that the high temperatures generated by the fault slip caused pyrite to transform into pyrrhotite at a transition temperature exceeding 500 °C^28^. Core drilling off the Pacific coast near the site of the Tohoku earthquake (2011) also determined that pyrite changed into pyrrhotite near the sliding surface and its corresponding transition temperature was greater than 640 °C^27^. Because pyrite or greigite was very stable up to 3.4 GPa, but the pressure of these fault-slips was less than 0.8 GPa and the duration for the fault-slip was very short (a few seconds or minutes, meaning the time effect is slight). Moreover, it is reported that the reaction of pyrite is primarily controlled by the temperature during the fault movement (pressure effect is minor)^27^. In this study, we observed via *in situ* XRD, that pyrite was completely transformed into pyrrhotite at temperatures above 580 °C. The TG-DTA results showed that pyrite transformed into pyrrhotite between 520 and 630 °C. Our phase transition temperature results were therefore similar to those that were found in nature (i.e., the fault-slip temperatures in the Chi-Chi and Tohoku earthquakes). Our study suggests that the phase transition temperature at which greigite undergoes phase transsition into pyrrhotite can provide an indicator of the fault-slip temperature.

## Conclusion

The temperature of a fault slip is difficult to determine. In this study, we synthesized greigite to investigate its high-temperature phase transitions and to simulate the temperature of fault movement. In the high-temperature phase transition process in an anaerobic environment, greigite started to transform into pyrite and pyrrhotite at 300 °C. At 600 °C, only the pyrrhotite phase was observed to remain. Our experimental results are similar to the observations made in the field, indicating that greigite can be used as an indicator of the fault-slip temperature.
